# The Rational Treatment of Nasal Catarrh

**Published:** 1886-09-20

**Authors:** A. G. Hobbs

**Affiliations:** Atlanta, Ga., Professor of Diseases of the Eye, Ear and Throat in Southern Medical College


### THE RATIONAL TREATMENT OF NASAL CATARRH.

By A. G. HOBBS, M. D., ATLANTA, GA.
**Professor of Diseases of the Eye, Ear and Throat in Southern Medical College.**

To treat successfully the diseases of the upper respiratory tract it is necessary that we have a full knowledge of the various means of examining the diseased parts, else our diagnosis will be guess-work, and our treatment as liable, if not more so, to do harm as to do good. With some physicians there seems to be but one name, and I might add, but one treatment for all diseases of the nose characterized by a discharge. It matters not whether the discharge be caused by a nasal polypus, a sarcomatous tumor, a fibroid, a foreign body, an ulceration, an hypertrophy over the turbinates, or an atrophy of the mucous membrane, it is all called *catarrh* and is treated in the same manner, by some so-called specific, by means of an anterior or posterior nasal douche or by the introduction of some irritating powder. These so-called specifics are always irritating and often very painful, so much so, that their use cannot be, and fortunately is not, persevered in.

The practitioner who invariably resorts to this mode of procedure in a routine way generally knows as little about what is producing the discharge as his unfortunate patient. Is it any wonder, then, that catarrh is regarded as incurable when it is so universally treated without any discrimination? Almost any medical journal we pick up contains some catarrh remedy vaunted as a *sure cure* without regard to the cause or character of the disease.

Since it is a fact that a large proportion of the communities in which we live suffer with some form of nasal catarrh, does it not seem strange that its pathology is not more generally studied, when the same amount of labor in this direction will give us better results than in almost any other towards an amelioration, and, in most cases, an entire cure of the disease? We have to treat the pathological conditions as we do in other diseases when we reach success. There can be no specific for a nasal discharge that may have so many causes.

If we would proceed rationally then in these diseases, the first thing we should do is to ascertain by a careful examination the cause of the discharge and the condition of the nasal membrane. If caused by a polypus, this must be extracted either with a snare or by the appliciation of chromic, acetic or nitric acid, or by the thermo-cautery. If it be caused by a fibroid or sarcomatous tumor the same means must be pursued, but either will be more difficult to extract than a polypus. If by a foreign body, it must be extracted. If by an ulceration, it must be locally cauterized and constitutionally treated, or both, as the case may demand. If hypertrophic tissue is found on the turbinates, it must be snared away if large, or scarified if it partakes of the nature of cavernous tissue. All of this should be done with practically no pain by the thorough use of cocaine.

In the meantime, what should be done in these cases or in others where no tumors are found? Should the anterior nasal douche be used containing some irritating astringent? Never! Because it does not reach any part of the nasal fossa except the floor, and while it may wash out some of the accumulations contained there its secondary action of causing the tissues to swell will overbalance the only good it can perform—that of washing the floor of the nose.

Should the posterior nasal douche be used? *Never!* While it can do no more good (if effectually used, and not one person in ten can ever learn to effectually use one) than the anterior douche it may do a great deal more harm by throwing the fluid into the eustachian tube and causing middle ear inflammation.

Then what is to be done when the catarrh is found to be due to an inflammation of the mucous lining of the nose (the result in nearly all cases of a succession of colds) without a polypus or a tumor, or a foreign body, or an ulceration?

Sprays containing some emolient and non-irritating fluid, such as wormed vaseline, furnish the only rational means of reaching every part of the diseased membrane for the purpose of cleansing and medicating without irritating it. It is necessary that the material used in the spray should be perfectly harmless to the healthy tissues, exert a soothing influence to the diseased tissues, and have a tendency to lessen the inflammation.

These sprays should be applied both posteriorly and anteriorly. After the parts are thoroughly cleansed any astringent, resolvent, or alterative, such as pinus canadensis, eucalyptol, sandlewood oil, wintergreen oil, pure terebene or carbolic acid, as the case may demand, may be added to the vaseline spray in small quantities.

It should be remembered, however, that no spray treatment should ever irritate the parts to which it is applied; hence the strength of the medicated spray should be gauged accordingly.

These treatments should be made daily by the physician himself for some weeks, then three times a week, and twice a week, according to the case. In this way alone do I believe that the great majority of nasal catarrhs can be cured.

As to the constitutional treatment, I do not believe any is necessary except as indicated by the systemic condition. A torpidity of the bowels or kidneys should of course have laxatives or diuretics. A weakened condition of the system will also demand tonics and stimulants, but in most cases no internal medication will be found necessary.

